# Syrian refugees’ experiences while receiving mental health services and psychiatric nursing care: A qualitative study

**DOI:** 10.1192/j.eurpsy.2024.1271

**Published:** 2024-08-27

**Authors:** G. Öztürk, K. Timarcioglu, G. Dikec, E. Karali, H. Nacaroglu, H. F. Cakir, A. K. H. Seren

**Affiliations:** ^1^Nursing, Fenerbahce University, Istanbul; ^2^Community Health Center, Turkish Red Cress, Sanliurfa, Türkiye; ^3^New Cross Hospital, 3Royal Wolverhampton Trust, Wolverhampton, United Kingdom; ^4^Community Health Center, Turkish Red Cress, Kahramanmaras; ^5^Disaster Risk Reduction, 4University of Health Sciences Mental Health and Neurological Diseases Education and Research Hospital, Istanbul, Türkiye

## Abstract

**Introduction:**

Millions of people have migrated because of violence, wars, disasters, and human rights violations, all of which have increased rapidly in recent years. Türkiye has hosted millions of refugees since 2010. Few studies have focused on the mental health needs of refugees or how these needs have been addressed in Türkiye.

**Objectives:**

This study examined the experiences of Syrian refugees in a community center in Turkiye as they access mental health services and receive psychiatric nursing care.

**Methods:**

A qualitative design was adopted in the study. Data were collected from southern Turkiye between November and December 2021. The researchers conducted three semistructured focus group interviews following Colaizzi’s phenomenological method to analyze the qualitative data. A total of 19 Syrian refugees participated in the focus group interviews.

**Results:**

Three key themes related to immigrants’ experiences of receiving mental health services and nursing care were identified: barriers to receiving mental health services, coping with negative experiences in Turkiye, and satisfaction with mental health services. The participants identified the barriers they experienced while receiving health services as those pertaining to language, discrimination, and stigmatization. They also mentioned the methods of coping with these negative experiences in Turkiye. Despite their negative experiences, they expressed satisfaction with the mental health services they received, especially psychiatric nursing care.

**Conclusions:**

This study determined that Syrian refugees face barriers to accessing and receiving mental health services. They stated that mental health professionals in Turkiye approach them with empathy, particularly those in psychiatric nursing. Healthcare professionals may be trained in culturally sensitive care to increase awareness. Studies have frequently examined the experiences of nurses providing care to refugees, but few have focused on evaluating nursing care from the perspective of refugees. Syrian refugees have reported various obstacles in accessing and receiving mental healthcare services. Health professionals, especially psychiatric nurses in mental health psychosocial support centers, must facilitate the processes to eliminate these obstacles.

**Disclosure of Interest:**

None Declared

